# TANGLE: Two-Level Support Vector Regression Approach for Protein Backbone Torsion Angle Prediction from Primary Sequences

**DOI:** 10.1371/journal.pone.0030361

**Published:** 2012-02-02

**Authors:** Jiangning Song, Hao Tan, Mingjun Wang, Geoffrey I. Webb, Tatsuya Akutsu

**Affiliations:** 1 Department of Biochemistry and Molecular Biology, Faculty of Medicine, Monash University, Melbourne, Victoria, Australia; 2 National Engineering Laboratory for Industrial Enzymes and Key Laboratory of Systems Microbial Biotechnology, Tianjin Institute of Industrial Biotechnology, Chinese Academy of Sciences, Tianjin, China; 3 Bioinformatics Center, Institute for Chemical Research, Kyoto University, Uji, Kyoto, Japan; 4 Faculty of Information Technology, Monash University, Melbourne, Victoria, Australia; Kyushu Institute of Technology, Japan

## Abstract

Protein backbone torsion angles (Phi) and (Psi) involve two rotation angles rotating around the C_α_-N bond (Phi) and the C_α_-C bond (Psi). Due to the planarity of the linked rigid peptide bonds, these two angles can essentially determine the backbone geometry of proteins. Accordingly, the accurate prediction of protein backbone torsion angle from sequence information can assist the prediction of protein structures. In this study, we develop a new approach called TANGLE (Torsion ANGLE predictor) to predict the protein backbone torsion angles from amino acid sequences. TANGLE uses a two-level support vector regression approach to perform real-value torsion angle prediction using a variety of features derived from amino acid sequences, including the evolutionary profiles in the form of position-specific scoring matrices, predicted secondary structure, solvent accessibility and natively disordered region as well as other global sequence features. When evaluated based on a large benchmark dataset of 1,526 non-homologous proteins, the mean absolute errors (MAEs) of the Phi and Psi angle prediction are 27.8° and 44.6°, respectively, which are 1% and 3% respectively lower than that using one of the state-of-the-art prediction tools ANGLOR. Moreover, the prediction of TANGLE is significantly better than a random predictor that was built on the amino acid-specific basis, with the *p*-value<1.46e-147 and 7.97e-150, respectively by the Wilcoxon signed rank test. As a complementary approach to the current torsion angle prediction algorithms, TANGLE should prove useful in predicting protein structural properties and assisting protein fold recognition by applying the predicted torsion angles as useful restraints. TANGLE is freely accessible at http://sunflower.kuicr.kyoto-u.ac.jp/~sjn/TANGLE/.

## Introduction

As a result of the completion of whole-genome sequencing projects, the sequence-structure gap is rapidly increasing. In this context, the accurate prediction of protein structure and function from sequences remains a challenging task. An useful intermediate way to address this is to predict one-dimensional structural properties of proteins including secondary structure, solvent accessibility, residue contact number/order, residue depth, and dihedral torsion angles [1]–[12]. For a comprehensive review of recent progress on the development of one-dimensional predictors, refer to Kurgan and Disfani [13]. In the past two decades, most efforts have been made to predict the former three properties of proteins, leading to ongoing improvements in prediction performance [14]–[16]. However, with respect to torsion angles, there is increasing interest in the field of structural bioinformatics in developing efficient algorithms that are capable of accurately predicting protein backbone torsion angles from amino acid sequences. This is because they can provide more detailed description of the backbone conformations, which, if known, can significantly reduce the conformational search and contribute towards the final prediction of protein three-dimensional structure predictions. For example, predicted torsion angles have been applied to improve protein secondary structure prediction [17], [18], protein fold recognition [19]–[21], multiple sequence alignments [22], [23] and fragment-free tertiary-structure prediction [10].

There are three different backbone torsion angles along with protein polypeptide chains: ϕ (Phi), ψ (Psi) and ω (Omega), which involve the backbone atoms C-N-C_α_-C, N-C_α_-C-N and C_α_-C-N-C_α_, respectively. Due to the planarity of the linked rigid peptide bonds, the two angles Phi and Psi can essentially determine the backbone geometry of proteins. The third angle Omega does not need to be specified as it is almost always fixed at 180° [11]. This means protein local structures can be unambiguously described by their backbone torsion angles [10]. Therefore, if the real values of Phi and Psi of all residues of a given protein are known, it will be more straightforward to re-construct the protein structure using the standard bond length [11]. In addition, protein backbone torsion angles are closely correlated with protein secondary structures [24]. Particularly, different secondary structure types are clustered in different regions in the Ramachandran Phi-Psi diagram [25], so it is therefore possible to predict protein secondary structures based on the predicted torsion angle probabilities. Accordingly, predicted torsion angles have been used as a replacement or supplement to secondary structure for refined local-structure predictions and have also been used to construct simplified protein models for sampling efficiency [9], [10].

Conventionally, torsion angles were predicted as a few discrete states based on the backbone conformation distributions and various computational algorithms were developed to predict the discrete states of Phi/Psi angle values [26]–[32]. Machine learning techniques are typically used to train and build prediction models, including neural networks [3], [11], [24], support vector machines [11], [24], [32] and hidden Markov models [28], [30]. In this direction, Helles and Fonseca have recently developed an artificial neural network framework to predict torsion angle probability distribution of coiled residues [33]. Their method achieved prediction accuracy comparable to that of secondary structure prediction (80%) and was significantly better (4–68%) than the baseline statistics. More recently, Kountouris and Hirst have created an SVM-based predictor called DISSPred of multi-state torsion angles and three-state secondary structures. It has achieved a more competitive predictive performance compared with other previously developed classifiers [34]. As a result of the free movement of proteins in the three-dimensional space, however, protein backbone torsion angles are actually continuously varying variables. Although these earlier methods have achieved prediction accuracy of up to 80% [24], [32], [34] based on the arbitrarily defined discrete states, such predictions cannot specify the actual Phi/Psi values for each state, and therefore have limited value in protein structure prediction.

In view of this, in recent years more attention has been given to real-value prediction of both Phi and Psi torsion angles. The first real-value prediction approach, DESTRUCT, was proposed by Wood and Hirst [35]. In their work, they used the PSI-BLAST program [36] to generate position-specific scoring matrices (PSSM), which was further taken as input to train the iterative neural network models and predict one of the two major torsion angles Psi. Nevertheless, the correlation coefficient between predicted and actual values of the Psi angles was only 0.47. Berjanskii *et al.* developed a web server, named PREDITOR for predicting protein torsion angles [37]. It combines sequence alignment methods with advanced chemical shift data to generate the predicted torsion angles. 88% of Phi/Psi predictions by PREDITOR are located within 30° of the correct values. Wu and Zhang proposed the ANGLOR predictor based on the composite machine-learning algorithm using support vector machines and neural networks, which has achieved a mean absolute error (MAE) of 28°/46° using built models trained on only 500 protein chains [11]. Dor and Zhou developed a method called Real-SPINE that predicts the real values of structural properties of proteins including residue solvent accessibility and backbone torsion angles, based on integrated neural networks [3]. Trained on a large dataset of 2,640 protein chains, Real-SPINE substantially improved the correlation coefficient to 0.62 between the predicted and actual Psi angles (10-fold cross-validation) through large-scale learning with a slow learning rate and over-fitting protection. Real-SPINE 2.0 server [12], Real-SPINE 3.0 [9] and SPINE X [10] were further developed by Zhou's group, with the prediction accuracy continuously improved by guided learning through neural networks and other refinement techniques. In addition, using a database of 997 non-redundant NMR structures, they have further developed a neural-network based predictor for the real-valued prediction of Phi and Psi angle fluctuations [38] based on sequence information only. This predictor achieved ten-fold cross-validated Pearson correlation coefficients (CC) of 0.59 and 0.60, and mean absolute errors of 22.7° and 24.3° for the angle fluctuation of ϕ and ψ, respectively [38]. Altogether, the consensus of these studies has been that real-valued torsion angle predictions by state-of-the-art algorithms have the potential to be employed as a replacement of or supplement to secondary-structure prediction tools, and are expected to substantially improve the quality of protein structure prediction when high-confidence predicted torsion angles are applied as constraints.

More recently, Ahmad *et al.* proposed a novel approach for the simultaneous prediction of eight one-dimensional structural features (including solvent accessibility, helix-helix contact and backbone torsion angles) for helical membrane proteins by using an integrated prediction system called HTM-One [39]. The performance of HTM-One has been shown to outperform respective models that were separately trained on individual features, which was evaluated using rigorous leave-one-out jackknife tests based on a non-redundant dataset of 286 helical membrane proteins [39]. The results indicate that compared with previous practice of training models individually, the performance of one-dimensional predictors can be significantly improved using this prediction system in an integrated manner. This is clearly an important step in the right direction for addressing the issue of how to improve the prediction performance of one-dimensional structural features of proteins from amino acid sequences.

In this study, we propose a new complementary approach to predict the Phi/Psi angles by support vector regression (SVR) learning from sequence information only. We want to take advantage of the excellent ability of SVR to generalize learning rules and predict the raw values of the given samples. The developed TANGLE (Torsion ANGLE) predictor works by integrating multiple local sequence profiles and global sequence features within a two-level SVR learning framework. Features used by TANGLE include multiple sequence alignment profiles retrieved from the position-specific scoring matrix (PSSM), predicted secondary structure, predicted solvent accessibility and predicted native disorder information. Moreover, other global sequence information such as amino acid contents, sequence length and sequence weight are used as the inputs to TANGLE. To improve the prediction accuracy, various combinations of different feature types with different local window sizes are systematically examined and compared. Finally, TANGLE achieves a significantly better prediction accuracy compared to the ANGLOR predictor [11] and a random amino acid-specific predictor when trained and evaluated on a large dataset with 1,989 protein chains. As an implementation of this approach, we have developed the TANGLE webserver for protein backbone torsion angle prediction. This is freely available at http://sunflower.kuicr.kyoto-u.ac.jp/~sjn/TANGLE/.

## Materials and Methods

### Datasets

In order to objectively compare our approach with other available approaches developed previously, we used the same datasets as originally developed by Wu and Zhang [11], where the PDB entries with any broken chains or missing residues were excluded. In this dataset, every two sequences in the dataset had a pair-wise sequence identity of less than 25%. Among them, 500 proteins were used as the training set, while the rest 1,026 proteins were used as the independent testing set. The total residues in the training and testing sets were 70,646 and 142,091, respectively.

The experimental values of Phi and Psi torsion angles were calculated by the DSSP program [40]. Because the four residues in the N- and C-terminus lacked four consecutive atoms that were required to form the torsion angles, they were neglected and not included in the prediction analysis. The calculated Phi/Psi angles by DSSP can be downloaded from our TANGLE website: http://sunflower.kuicr.kyoto-u.ac.jp/~sjn/TANGLE/links.

We normalized the original Phi and Psi angles using their average and standard deviations based on the whole training datasets, to make most of their values fall within the range between 0 and 1, as suggested previously [5]–[7]. In the training stage, the prediction models were trained based on the normalized values of Phi and Psi, instead of the original values. In the prediction stage, we first predicted the normalized Phi and Psi angles from primary sequences in the independent test set, and then recovered the absolute Phi and Psi angles from their respectively predicted normalized values. The calculated Phi and Psi angles in the training set of 500 proteins chains can be found in Datasets S1 and S2, respectively, while the calculated Phi and Psi angles in the testing set of 1,026 protein chains can be found in Datasets S3 and S4, respectively.

### Performance Evaluation

To measure the performance of real-valued torsion angle predictions, we calculated three different measures, the Pearson correlation coefficient, the mean absolute error and root mean square error between predicted and observed Phi and Psi torsion angles.

The Pearson's correlation coefficient (CC) between the predicted and observed torsion angle values is defined as:
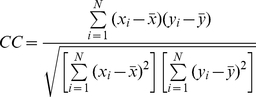
(1)where *x*_i_ and *y*_i_ are the observed and predicted torsion angle values of the *i*-th residue, respectively, 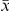 and 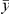 are their corresponding means and *N* is the total number of residues in a protein sequence. CC = 1 indicates that the two sets of values are fully correlated, while CC = 0 indicates that they are completely uncorrelated.

The mean absolute error (MAE) is defined as the average difference in angle degrees between the predicted and the observed torsion angles of all residues, i.e.
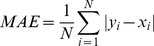
(2)


The root mean square error (RMSE) is given by:
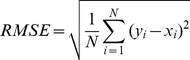
(3)Two RMSE measures were calculated in this study: RMSE_norm and RMSE_raw. The former was calculated based on the normalized values of Phi/Psi angles, while the latter was calculated based on the original (raw) values of Phi/Psi angles. In addition, the CC, RMSE_norm, RMSE_raw and MAE measures were calculated on both the protein chain and residue level, respectively.

### Support vector regression (SVR)

Support vector machine (SVM) is a sophisticated supervised machine learning technique based on statistical learning theory [41], [42]. SVM is especially effective when the input data is not linearly separable and the kernel function is required to map the data into a higher dimensional space to find the optimal separating hyperplane. In practice, SVM has two modes: support vector classification (SVC) and support vector regression (SVR). Due to its excellent regression ability, SVR has been applied to predicting accessible surface area [43], contact number [5], [44], B-factor [45], residue depth [8], disulfide connectivity [46], caspase cleavage site [47], gene expression level [48], missing value estimation in microarray data [49], peptide-MHC binding affinity [50], siRNA efficacy [51], gene selection [52], domain boundary [53], and antigenic epitope [54].

In the present study, we use SVR (implemented in the SVM_light package, available at http://svmlight.joachims.org/) to predict torsion angle values from amino acid sequences. We selected radial basis kernel function (RBF) at ε = 0.01, γ = 0.01 and C = 5.0 to build the models for both the first-level and second-level SVR in TANGLE. This combination of parameters has been shown to provide the best prediction performance in the preliminary analysis through selecting and comparing different combinations of C and ε and examining their respective prediction performances. In the following analysis, we constantly set ε as 0.01, γ as 0.01 and C as 5.0 to evaluate the prediction performance of other sequence encoding schemes. Selection of SVM parameters and features using a sliding window size were done using only the training dataset.

### Two-level support vector regression approach of TANGLE

In this section, we will describe the design of our two-level TANGLE approach that uses two SVR predictors in cascade for predicting protein backbone torsion angles from protein primary sequences. In TANGLE, the first-level accepts all the sequence-derived features as inputs to SVR and outputs the initially predicted torsion angles. The second-level accepts the initially predicted torsion angles by the first-level SVR predictor and outputs the final refined torsion angles. As the torsion angles of a residue at a particular position in the sequence depend on the local structure of its neighboring residues, introducing another layer of SVR predictor that incorporates the contextual relationship of torsion angles in the proximal neighborhood can potentially enhance the torsion angle prediction of that residue [55]. The idea of designing a two-level SVR approach has been proposed in previous studies of predicting protein solvent accessibility [55]–[57], residue B-factors [58], as well as analyzing condition-specific regulatory networks [59], where use of two-level SVR has been demonstrated to improve the robustness of the prediction system and enhance prediction accuracy.

**Figure 1:**
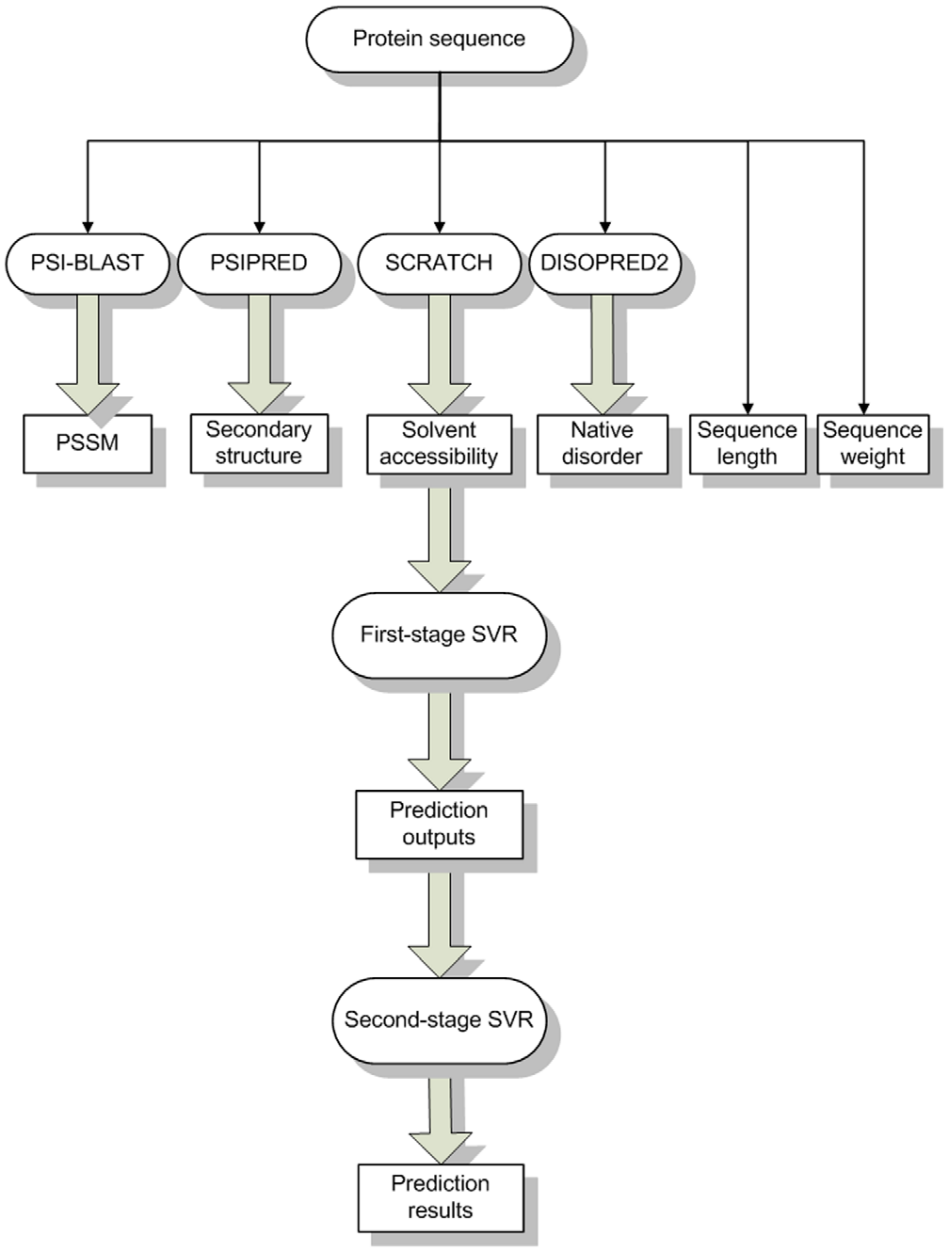
The architecture of TANGLE for protein backbone Phi and Psi angle predictions. Six different types of sequence and structural features are generated and used as input to build the two-level SVR models of TANGLE. These features include position-specific scoring matrix (PSSM), PSIPRED-predicted secondary structure, SCRATCH-predicted solvent accessibility, DISOPRED2-predicted native disorder, sequence length and sequence weight.

In this study, we are interested in investigating the influence of various sequence features and their combinations on the prediction performance of torsion angles, within the two-level SVR framework. Figure 1 illustrates the flowchart of our two-level TANGLE approach. As can be seen, there are six different types of sequence-derived features that will be used as inputs to the first-level SVR. These features include (1) position-specific scoring matrices (PSSM) [36]; (2) PSIPRED-predicted secondary structure [60]; (3) SCRATCH-predicted solvent accessibility [61]; (4) DISOPRED2-predicted native disorder [62] and two other global features including (5) sequence length and (6) sequence weight [5]–[8]. Detailed description of these features and their extraction and encoding procedures are provided in the following “Sequence encoding schemes” Section.

The second-level SVR takes the predicted output of the first-level SVR with the purpose to further enhance the prediction of torsion angles. Previous studies have indicated that the use of a second-level SVR in cascade can improve the prediction accuracy by capturing the contextual relationships underlying protein structural property values like solvent accessibility and B-factors from the output of the first-level SVR [55]–[58]. Notice that in both in the first- and second-level SVR predictors, the sequence features for a residue of interest are encoded into input vectors of SVR using a sliding local window approach. This will be briefly discussed in the following section.

### Sequence encoding schemes

Selecting appropriate sequence encoding schemes is an important step as it determines the quality of feature extraction of SVR models and thus has a significant impact on the prediction performance. In this section, we describe in more detail how to extract and encode different types of sequence feature.

### Position-specific scoring matrices (PSSMs) in the form of PSI-BLAST profiles

Position-specific scoring matrix (PSSM) of a residue in the form of PSI-BLAST profile contains important evolutionary information that determines whether this residue is conserved in its family of related proteins. Each element in the PSSM represents the probability of each residue position in the multiple sequence alignment. Numerous previous studies have shown that multiple sequence alignments in the form of position-specific scoring matrices (PSSMs) can significantly improve overall prediction performance [63]–[77].

**Figure 2:**
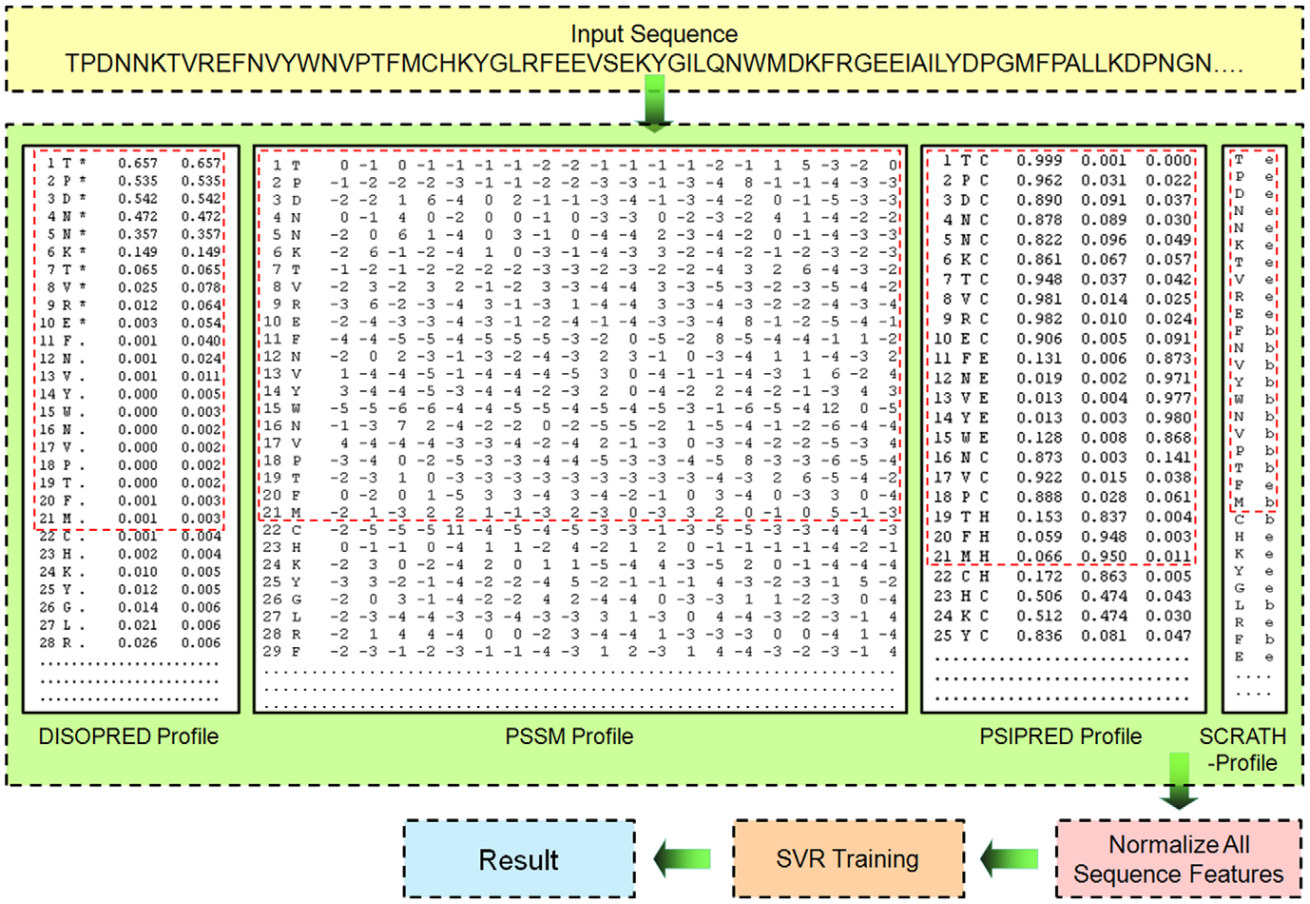
A sliding window approach is employed to extract and encode local profiles into the first-level SVR model of TANGLE. The sequence-encoding scheme “PB+PP+SC+DISO” is taken as an example to illustrate how to extract the local profiles. Here, window size *L* is set up at *L* = 21 for a residue of interest (Residue F, position 11 in this example).

In this study, we obtained the PSSM profile for each sequence in the datasets by running PSI-BLAST search and encoded each residue using a local sliding window approach based on the PSSM profiles. PSI-BLAST was run for three iterations against the non-redundant NCBI nr database using a default *E*-value cutoff to obtain the PSSMs profiles. All the elements in the PSSM profiles were divided by 10 for normalization, so that most of the values fell with the range of 0 and 1. For a given residue, its local sequence fragment was extracted and encoded as a 20×(2*l*+1)-dimensional vector using a sliding window scheme where *l* denotes the half window size and *L* = 2*l*+1 is the full window length (See Figure 2 for extraction and encoding). In order to select the optimal local window size *L* for the Phi and Psi angle prediction, we evaluated prediction performance of a variety of different local window sizes *L*, ranging from 3 to 21. In summary, in this encoding scheme, a residue was encoded by a 20×*L* = 20×(2*l*+1)-dimensional vector.

### Predicted secondary structure information by PSIPRED

The PSIPRED program was chosen to predict the secondary structure information. PSIPRED is an accurate neural network-based predictor for the prediction of secondary structure with an accuracy of up to 80% [60]. The output of PSIPRED includes three-state (helix/strand/loop) prediction and probability scores for each secondary structure type. The users can submit a protein sequence and receive the prediction result both textually via e-mail and graphically via the webserver. In our previous work, we have shown that incorporation of PSIPRED-predicted secondary structure information can significantly improve the prediction performance [6]–[8].

Similarly, for a given residue, its three-state secondary structure profile was extracted and encoded using a sliding window of *L* = 2*l*+1 (*l* = 1, 2, 3, …, 10) consecutive residues. Therefore, in this encoding scheme, a residue was encoded by a 3×*L* = 3×(2*l*+1)-dimensional vector.

### Predicted solvent accessibility information by SCRATCH

The SSpro program in the SCRATCH software package [61] was used to predict the solvent accessibility of each residue in the datasets. SSpro yields the predicted solvent accessibility status for a residue, in a binary format- either as “exposed” or “buried”. The predicted solvent accessibility has been shown to be able to improve the prediction accuracy for predicting natively unstructured regions [78], [79] or loops [80], DNA-binding sites [66], as well as protein interaction hotspots [67]. In this encoding scheme, a residue was encoded by a 2×*L* = 2×(2*l*+1)-dimensional vector.

### Predicted native disorder information by DISOPRED2

In recent years, researchers have realized that natively disordered regions are commonly responsible for important protein function. As such, there has been an increasing interest in studying such regions in proteins. Natively disordered or unstructured regions are found to be associated with molecular assembly, protein modification and molecular recognition [81]–[83]. Therefore, inclusion of this feature into the SVR models could potentially improve the performance of torsion angle prediction. In previous work, native disorder features have been used to enhance the prediction performance on caspase cleavage sites [46] and phosphorylation sites [84].

In this study, we used the DISOPRED2 server, which was developed using neural networks and is considered to be one of the best predictors for predicting natively unstructured or disordered region [62]. DISOPRED2 outputs the predicted possibility of each residue being natively disordered or ordered, which will be extracted and input into the SVR models. In this encoding scheme, a residue was encoded by a 2×*L* = 2×(2*l*+1)-dimensional vector.

### Other global sequence features

In addition to the sequence and structural features discussed above, we also included some representative global sequence features like the compositions of twenty amino acids, sequence length and sequence weight (Figure 1) and incorporated them into the SVR models of TANGLE. These complement local features. Previous studies have indicated that inclusion of these global sequence features can help to further improve prediction performance in a number of different real-value prediction tasks, i.e. prediction of residue contact number [5], residue-contact order [7], disulfide connectivity pattern [46], half-sphere exposure [6] and residue depth [8]. Incorporation of these global features has been shown to be helpful for improving the prediction performance [6]–[8].

To comprehensively investigate the influence of each feature type and improve the prediction performance, we train SVR models using six different sequence encoding schemes. For brevity, we refer to the encoding schemes based on PSI-BLAST profile, PSIPRED-predicted secondary structure, SCRATCH-predicted solvent accessibility, DISOPRED-predicted native disorder and all the combined sequence features, as ‘PB’, ‘PP’, ‘SC’, ‘DISO’ and ‘ALL’, respectively. With the increasing complexity of considered features, the dimensionality of input vector will increase accordingly. In the case of sequence encoding scheme “PB+PP+SC+DISO”, the total number of vector dimension is (20×*L*+3×L+2×L+2×L) = 27*L*. For example, for a local window size of *L* = 9, there are in total 243-dimensional vector designed to characterize each residue.

### The Sliding window approach to extract the local sequence and structural profiles

For residue encoding, a sliding window approach was used to extract the local sequence profile of each residue in the datasets. For sequence encoding schemes based on feature combinations, the extracted local profiles of various feature types will be further concatenated to generate the SVR inputs. Figure 2 illustrates how to extract local sequence profiles using this sliding window approach in TANGLE, taking sequence encoding scheme “PB+PP+SC+DISO” as an example.

## Results

### Statistical distribution of Phi and Psi angles

**Figure 3:**
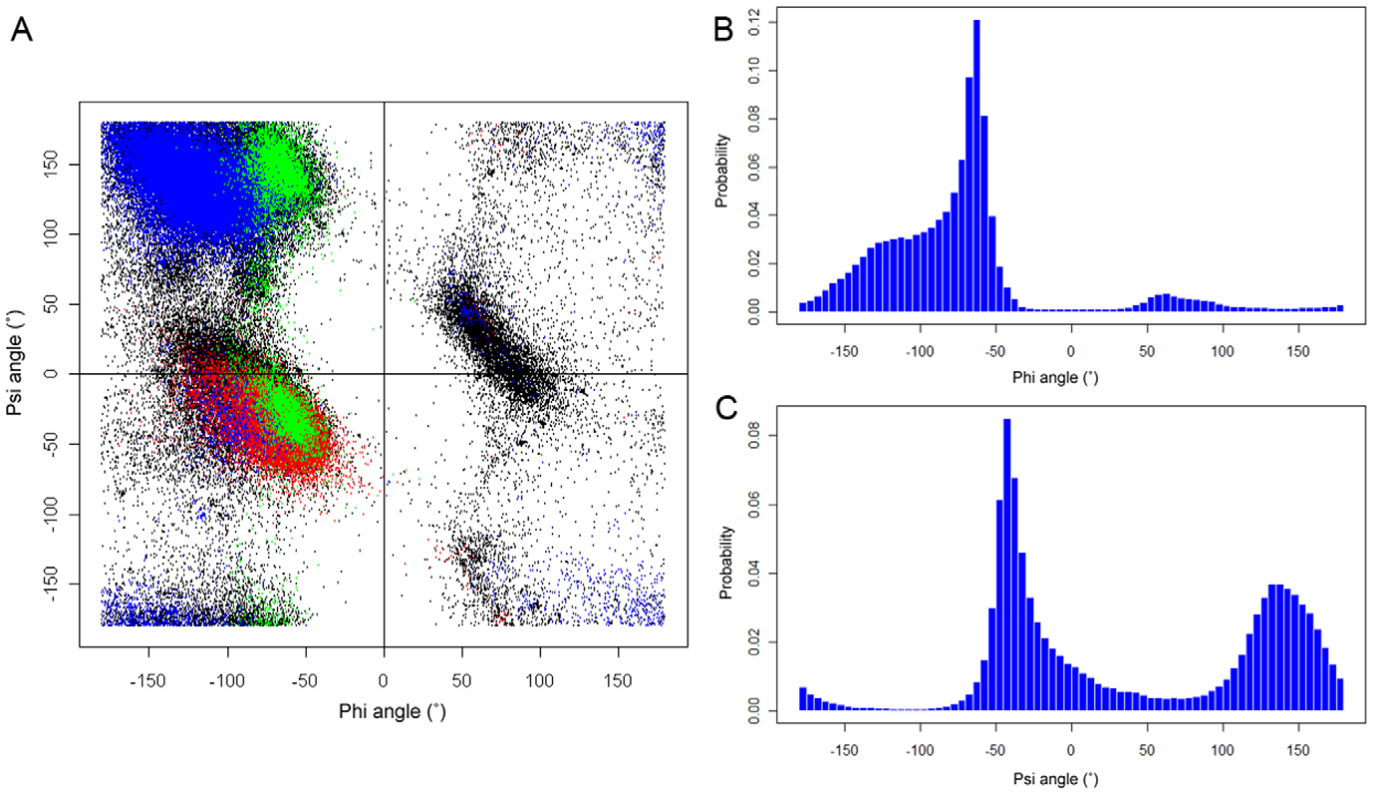
The Ramachandran plot and histogram distributions of Phi and Psi angles for all residues in the training set of 500 proteins. (A) The Ramachandran plot; (B) histogram of Phi angles; (C) histogram of Psi angles. Alpha-helix, beta-strand, proline and coil are represented by red, blue, green and black, respectively.

The distribution of Phi and Psi angles are displayed using the Ramachandran plot, as shown in Figure 3. This distribution is calculated using the training set with 500 PDB structures containing 70,646 residues. It is apparent that Phi and Psi angles have different distribution patterns: the former only has one peak around −70°, while the latter has two peaks around −50° and 130°, respectively. As discussed previously, the single-peak distribution of phi angles and double-peak distribution of psi angles in the Ramachandran plot, result in the different degrees of uncertainty and therefore the different prediction accuracy for the phi and psi angles [11]. This leads to different prediction difficulty for these two types of torsion angles. Due to their double-peak distribution, it is more difficult to predict Psi angles than the single-peak Phi angles, which is reflected by higher MAE and RMSE values for Phi angles but lower values for Psi angles.

The distribution of Phi/Psi torsion angles shows strikingly different patterns between different secondary structure types. As can be seen from Figure 3, most residues in alpha-helices are located within a narrow range of Phi and Psi angles. The populated area of alpha-helix residues is in the range of −150°<Phi<−20° and −100°<Psi<45°. While in the case of beta-strand residues, the two most populated areas are in the range of −150°<Phi<−20° and −100°<Psi<45°, and the range of −150°<Phi<−20° and −100°<Psi<45°, respectively. In contrast to alpha-helix and beta-strand residues, coil residues populate a much broader and diverse area, indicating that torsion angles of coil residues are very flexible and there are no apparent recurrent patterns like those in alpha-helices and beta-strands. This makes it more difficult to predict their Phi and Psi angles [33]. In the case of proline residues, the majority of them are found in the most populated area with torsion angles (Phi, Psi) of roughly roughly (−75°, 150°), corresponding to polyproline II helix. In summary, the distribution patterns of torsion angles reflect their roles of internal steric constraints that form different types of secondary structures.

### Effect of different local window size on the prediction performance

In this section, we chose different local window sizes and calculated the resulting prediction performance in order to examine the effect of various local window sizes using PSI-BLAST profiles. The performance achieved is shown in Table 1. As increasing the local window size provides more local information, it is reasonable to expect that prediction performance would increase with the enlargement of the window size. It is also expected that prediction performance would begin to decrease beyond a certain window size, as increasing the local window size also leads to the inclusion of more noise on the other hand. From Table 1, we find that this is indeed the case. At a local window size *L* = 9, the SVR model achieved the best prediction performance for the Phi angle prediction, with a CC of 0.486 and MAE of 29.92. In the case of Psi angle prediction, using local window size *L* = 13 led to the best prediction accuracy of CC = 0.581 and MAE = 55.38. However, *L* = 9, 11 and 13 have very similar effect on the prediction performance in terms of CC, RMSE and MAE measures. Consequently, in the following analysis, we selected all the three window sizes for comparing the performance of different sequence encoding schemes.

**Table 1:** Predictive performance of Phi and Psi angles based on different local window sizes using the PSI-BLAST profile.

Torsion angles	Local window size	Number of features	Number of support vectors	CC	RMSE	MAE
Phi	3	60	69370	0.455	49.25	31.44
	5	100	69358	0.478	48.57	30.44
	7	140	69299	0.484	48.33	30.05
	**9**	**180**	**69285**	**0.486**	**48.24**	**29.92**
	11	220	69243	0.483	48.27	29.94
	13	260	69343	0.478	48.42	30.04
	15	300	69382	0.472	48.55	30.25
	17	340	69350	0.466	48.73	30.46
	19	380	69369	0.459	48.90	30.71
	21	420	69344	0.451	49.13	30.99
Psi	3	60	69955	0.469	80.79	63.33
	5	100	69923	0.537	76.85	58.84
	7	140	69855	0.563	75.27	56.91
	9	180	69712	0.575	74.55	55.96
	11	220	69738	0.581	74.18	55.43
	**13**	**260**	**69718**	**0.581**	**74.24**	**55.38**
	15	300	69736	0.580	74.44	55.43
	17	340	69724	0.577	74.70	55.68
	19	380	69719	0.573	75.08	56.04
	21	420	69665	0.569	75.43	56.41

The results were obtained using an independent test set of 1,026 proteins from the set of PDB data compiled by Wu and Zhang [11], where the rest 500 proteins were used for training.

### Effect of different sequence encoding schemes on the predictive performance

Based on the extracted sequence and predicted structural profiles, we further developed two-level SVR models using different combinations of these profile features, as described in the Methods Section. The prediction performance of Phi and Psi angles by this two-level TANGLE approach on the testing set of 1,026 proteins can be found in Datasets S5 and S6, respectively.

Table 2 compares the prediction performance between six different sequence encoding schemes on the testing dataset with 1,026 protein chains. As shown in Table 2, we see that the sequence encoding scheme “PB+PP” that combines evolutionary information in the form of PSI-BLAST profiles (“PB”) along with predicted secondary structure information by PSIPRED (“PP”) achieved the best overall results for Phi angle prediction. The TANGLE model based on this encoding scheme achieved an overall CC of 0.529, RMSE of 46.72 and MAE of 27.85. This is better than other sequence encoding schemes. In addition, another two sequence encoding schemes “PB+PP+SC” and “PB+PP+DISO” achieved similar results, with the same CC values of 0.528, and slightly different MAE values of 27.87 and 27.89, respectively. These results, however, are slightly worse than the best sequence encoding scheme “PB+PP”.

**Table 2:** Prediction performance of Phi and Psi angles using the SVR predictors based on eight different sequence encoding schemes that incorporate various combinations of different types of sequence and structural features.

Torsion angles	Sequence encoding schemes	Number of features	Number of support vectors	Window Size	CC	RMSE	MAE
Phi	PB	180	69284	9	0.486	48.24	29.92
		220	69242	11	0.483	48.27	29.94
		260	69342	13	0.478	48.42	30.04
	PB+PP	**207**	**68913**	**9**	**0.529**	**46.72**	**27.85**
		253	68982	11	0.524	46.88	28.08
		299	69040	13	0.518	47.08	28.35
	PB+PP+SC	225	68948	9	0.528	46.74	27.87
		275	69041	11	0.522	46.94	28.18
		325	69096	13	0.515	47.17	28.52
	PB+PP+DISO	225	68928	9	0.528	46.72	27.89
		275	69054	11	0.523	46.91	28.13
		325	69082	13	0.516	47.14	28.44
	PB+PP+SC+DISO	243	69075	9	0.527	46.78	27.92
		297	69098	11	0.52	47	28.25
		351	69163	13	0.513	47.24	28.63
	ALL	277	68929	9	0.525	46.82	27.99
		331	69019	11	0.518	47.05	28.33
		385	69099	13	0.511	47.31	28.71
Psi	PB	180	69711	9	0.575	74.55	55.97
		220	69737	11	0.581	74.18	55.43
		260	69717	13	0.581	74.25	55.39
	PB+PP	207	68613	9	0.652	69.61	44.72
		253	68743	11	0.65	69.75	45.09
		299	68772	13	0.648	69.99	45.64
	PB+PP+SC	**225**	**68672**	**9**	**0.654**	**69.45**	**44.64**
		275	68857	11	0.652	69.65	45.15
		325	68961	13	0.649	69.94	45.79
	PB+PP+DISO	225	68704	9	0.649	69.94	45.79
		275	68811	11	0.65	69.82	45.24
		325	68866	13	0.647	70.07	45.84
	PB+PP+SC+DISO	243	68681	9	0.653	69.51	44.73
		297	68805	11	0.651	69.73	45.29
		351	68971	13	0.648	70.03	46.00
	ALL	277	68779	9	0.654	69.48	44.82
		331	68854	11	0.652	69.68	45.38
		385	68977	13	0.648	70.02	46.10

Prediction performance of three different window sizes *L* = 9, 11 and 13 is provided. The results were obtained using an independent test set of 1,026 proteins from the set of PDB data compiled by Wu and Zhang [11], where the rest 500 proteins were used for training.

For Psi angle prediction, the sequence-encoding scheme “PB+PP+SC” that integrates the PSI-BLAST profile with predicted secondary structure and solvent accessibility information, achieved the best overall results. This encoding scheme achieved CC of 0.654, RMSE of 69.45 and MAE of 44.64 between the predicted and observed Phi angles (Table 2). These results suggest that using predicted secondary structure information in combination with PSI-BLAST profiles greatly enhanced the prediction of Phi and Psi torsion angles, which is reasonable considering that there are strong correlations between torsion angle distribution and regular secondary structure types such as alpha-helices and beta-strands. In addition, compared with Phi angle, higher RMSE and MAE values of Psi angle prediction again confirm that they are more difficult to predict.

We further incorporated the predicted solvent accessibility profile (“SC”) into the two-level SVR models. We found that usage of this information is particularly helpful for improving the prediction performance of Psi angles. However, it is not very useful for Phi angle prediction. We also investigate whether inclusion of predicted native disorder information (“DISO”) would further improve the prediction performance of torsion angles. It is somewhat surprising to see that usage of this information actually decreases the prediction accuracy, as reflected by lower CC and higher MAE values after incorporation of such features into two-level SVR models. This suggests that the predicted native disorder profile is not helpful in improving the prediction quality of the Phi/Psi angles.

**Figure 4:**
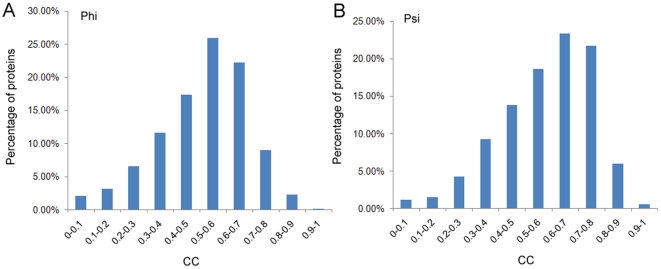
The distributions of correlation coefficients (CCs) of the Phi and Psi angle prediction for 1,026 protein chains in the testing dataset.

**Figure 5:**
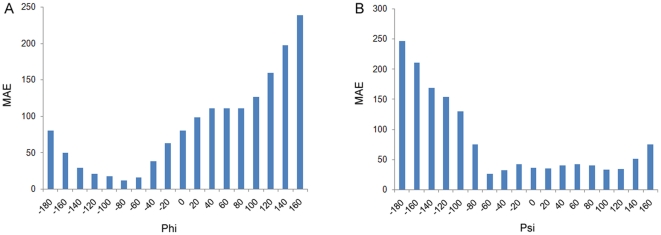
The mean absolute errors (MAEs) between the predicted and observed Phi and Psi angles, as a function of the observed angles, divided into bins with equal size of 20°.

To measure the prediction performance at the protein chain level, we calculated the CCs between the predicted and observed Phi/Psi angles for each protein chain in the testing dataset, as shown in Figure 4. We can see that more than 50% of protein chains have a CC of 0.6 or more, and no less than 70% of proteins have CC of at least 0.5. We further analyzed the distribution of MAEs that were averaged on each protein chain, in relation to the observed Phi/Psi angles. This is shown in Figure 5. We can see that residues with Phi angles in the range of 100° to 160° and residues with Psi angles in the range of −180° to −100° have relatively large MAEs, indicating that the predicted Phi/Psi angels for these residues have greater errors. This is both because higher magnitude values will tend to have higher magnitude MAEs and because these residues are under-represented in the current datasets. It is also due to the fact that the SVR models cannot be well trained given that inadequate numbers of data points are fed into SVR. In comparison, residues in the most populated areas in the Ramachandran plot (Figure 3) have the smallest MAEs, e.g. those with Phi angles in the range of −140° to −60° and those with Psi angles in the range of −60° to 120° (Figure 5).

In Table 3, we provided the MAEs of Phi/Psi angle prediction results for residues according to twenty residue, three secondary structure and two-state solvent accessibility types. It is generally accepted that that coils are much more flexible and tend to adopt a greater variability of torsion angles. Accordingly, the MAE values of the coil residues are much higher than that of alpha-helix and beta-strand residues (Table 3). Overall, alpha-helix residues have the smallest MAEs (9.9° for Phi and 18.7° for Psi angle), while coil residues have the largest MAE values (40.8° for Phi and 66.0° for Psi angle). The difficulty of torsion angle prediction for different secondary structure types, as evaluated by MAE values, is closely related with the complexities of the torsion angle distribution (Figure 3) [11].

**Table 3:** Prediction performance comparison of TANGLE with ANGLOR and the random amino acid-specific predictor.

		Phi angle (°)			Psi angle (°)		
		MAE_TANGLE_	MAE_ANGLOR_	MAE_random_	MAE_TANGLE_	MAE_ANGLOR_	MAE_random_
AA^a^	ALA	21.9	22.5	27.4	38.2	42.7	79.7
	CYS	25.5	27.7	32.5	45.0	48.7	85.3
	ASP	29.7	30.8	32.2	48.7	48.9	73.5
	GLU	22.3	23.3	27.4	39.1	43.1	75.2
	PHE	23.6	24.2	32.0	39.4	40.8	85.8
	GLY	84.1	75.1	95.1	76.7	66.9	79.2
	HIS	29.6	31.8	35.7	46.4	48.2	76.4
	ILE	17.5	18.1	26.4	32.1	35.3	84.4
	LYS	24.8	25.6	30.6	41.8	45.6	79.0
	LEU	17.8	18.3	24.4	35.2	38.1	81.4
	MET	22.0	22.4	29.5	36.5	40.9	81.6
	ASN	37.1	37.6	42.3	45.2	45.9	68.2
	PRO	13.6	15.2	19.5	59.3	61.3	86.3
	GLN	23.9	25.1	30.0	39.4	43.0	76.9
	ARG	23.5	25.0	30.4	40.9	44.1	80.5
	SER	30.6	32.3	35.3	53.5	55.4	87.0
	THR	23.9	26.0	29.9	50.4	51.1	88.6
	VAL	19.1	20.1	28.5	34.8	37.6	83.1
	TRP	22.8	23.1	29.8	41.6	43.5	86.4
	TYR	23.7	25.3	32.4	40.1	42.3	85.5
All		27.8	28.2	33.8	44.6	46.4	80.9
SS^b^	H	9.9	11.0	19.0	18.7	28.2	29.3
	E	26.1	27.9	28.1	38.9	39.9	36.1
	C	40.8	41.8	51.5	66.0	63.9	81.3
SA^c^	E	30.7	31.2	55.7	47.0	49.9	84.6
	B	24.1	24.1	52.0	40.2	41.5	84.0

Prediction performance is categorized according to twenty amino acid types, three secondary structure types (H, helix; E, beta-strand; and C, coil) and two-state solvent accessibility (E, exposed and B, buried), evaluated by the mean absolute error (MAE). The results were obtained using an independent test set of 1,026 proteins from the set of PDB data compiled by Wu and Zhang [11], where the rest 500 proteins were used for training.

a Twenty amino acid types.

b Three secondary structure types. H: alpha-helix; E: beta-strand; C: coil.

c Two-class solvent accessibility: E: exposed; B: buried.

Moreover, because of the various degrees of steric collisions between the side-chain and main-chain of different amino acids, it is expected that different amino acid types have different levels of MAEs. In turn, this could reflect the various degree of difficulty for torsion angle predictions [11]. Taking this into consideration, we examined the prediction performance of TANGLE for twenty amino acid types and calculated their MAE values, as shown in Table 3. Among them, glycine has the largest prediction error, with MAE of 84° for Phi and 77° for Psi, respectively. This is not surprising because glycine has no side chain atom except for a proton, meaning that this amino acid has little geometrical restriction to its backbone torsion angle rotations. Proline is a special amino acid due to the presence of a distinctive cyclic structure in its side chain. Its Phi angle, which is almost locked at approximately −75°, restricts the backbone rotation in the direction of Phi angle. This gives proline an exceptional conformational rigidity compared to other amino acids. On the other hand, because it does not have an amide proton, the inclination of its side-chain towards the nitrogen atom results in nearly no steric restriction in the direction of Psi angle. As a result, proline has the least MAE error for Phi angle (13.6°), but the second largest MAE of 59° for Psi angle.

We further divided the residues into two types (buried or exposed) according to the conventional two-state solvent accessibility. The assignment of two-state solvent accessibility was based on the prediction results by the SCRATCH program [61]. From Table 3, we found that the buried residues have relatively smaller MAE values (24.1° for Phi and 40.2° for Psi, respectively) than exposed residues (30.7° for Phi and 47.0° for Psi, respectively). This indicates that the torsion angles of the exposed residues are more difficult to predict than the buried residues. It is worth mentioning that this result is consistent with previous work [11]. The reason might be that residues buried in the core regions of protein structures have less flexibility and more rigid structural constraints compared with exposed residues located on protein surfaces.

### Performance comparison with other approaches

The work that was most closely related to the present study was recently developed by Wu and Zhang, who presented a neural network and support vector machine-based predictor called ANGLOR to predict real values of torsion angles from primary sequences [11]. We compared the prediction performance of our TANGLE predictor with ANGLOR. This is a predictor built using support vector machines and neural networks, based on three different types of sequence-derived features including position-specific scoring matrices (PSSMs), predicted secondary structure and solvent accessibility information.

Another state-of-the-art predictor HTM-One is an integrated model that was specifically developed to predict eight one-dimensional structural features (including Phi and Psi torsion angles) for membrane proteins only [39], while TANGLE is a two-stage model that was trained to predict protein backbone torsion angles. Due to the different properties of membrane proteins, it is infeasible to make a fair comparison of the predictive capabilities of HTM-One and TANLGE. In terms of the advantages and disadvantages of integrated model versus two-stage model, the integrated model is more likely to avoid overfitting because it uses various kinds of training data. Further, the integrated model may be particularly useful when the availability of protein data is limited because it can use various features for training. However, in the case of two-stage model learning using SVM or SVR, it is difficult to use SVM or SVR for integrated model learning because standard SVM/SVR is designed for prediction of a single feature. Thus, it is difficult to apply the integrated approach to solve problems for which SVM/SVR is very useful.

We note that rigorous comparison with other available tools is meaningful only when they are developed and tested based on the same training and testing datasets. As we used exactly the same training dataset and testing dataset as the ones used in developing ANGLOR, we could directly make a performance comparison between the two tools. In addition, we also compared TANGLE with a random amino acid-specific predictor, which was built by randomly assigning the Phi/Psi angles to a residue from amino acid-specific pool collected from 500 protein chains in the training dataset, as suggested by [11]. Intuitively, this amino-acid-specific random predictor is able to provide more accurate torsion angle prediction than a complete random predictor which did not take into account amino acid type information. The randomization process for assigning Phi/Psi angles for each predicted residue in the testing dataset of 1,026 protein chains is repeated 10,000 times to achieve a stable predicted angle distribution [11]. The performance comparison between these three predictors is presented in Table 3.

Overall, for Phi angle prediction, the performance of TANGLE is higher (with MAE = 27.8° for all residues) than that of the random amino acid-specific (with MAE = 33.8° for all residues) and also outperforms ANGLOR (with MAE = 28.2° for all residues). In particular, the prediction of TANGLE is significantly better than a random predictor that was built on the amino acid-specific basis, with the *p*-value <1.46e-147 and 7.97e-150 for Phi and Psi angle prediction, respectively, by the Wilcoxon signed rank test. In contrast to the Phi prediction, the Psi prediction accuracy of TANGLE (with MAE = 44.6° for all residues) is significantly higher than that of the random amino acid-specific predictor (with MAE = 80.9° for all residues) and also higher than that of ANGLOR predictor (with MAE = 46.4° for all residues). At specific amino acid residue level, the MAE of TANGLE is significantly smaller than that of the random predictor for all the twenty amino acid types. At the second structure level, the MAE of TANGLE is also smaller than the random predictor for all the three-second structure types.

Compared with ANGLOR, the MAE of TANGLE is smaller than that of the ANGLOR predictor in terms of both Phi and Psi angle prediction, except for glycine, for which the MAE of TANGLE (84.1° for Phi and 76.7° for Psi) is higher than that of ANGLOR (75.1° for Phi and 66.9° for Psi). The improvement of real-value prediction of torsion angles by TANGLE can be attributed to a combination of multiple factors. While ANGLOR used neural networks to train the predictors for Phi angle prediction and SVM and three types of sequence-based features to train the models for Psi angle prediction, TANGLE used a two-level support vector regression system to refine the prediction results, based on more integrated multiple sequence and predicted structural features. In addition to the difference of optimal local window sizes used by the two predictors, the performance improvement may be attributed to the design and implementation of the two-level support vector regression-learning framework in TANGLE.

### The TANGLE server

For the implementation of this work, we have constructed an online server to provide a free academic service of torsion angle prediction from primary sequences, which is available at http://sunflower.kuicr.kyoto-u.ac.jp/~sjn/TANGLE/webserver.html. TANGLE requires the user to submit a single amino acid sequence in the FASTA format of the query protein as input, and an Email address to send out the prediction result. When the query sequence is submitted, several third-party programs including PSI-BLAST, PSIPRED, SCRATCH and DISOPRED2 will be executed to generate the respective PSSM, predicted secondary structure, solvent accessibility and native disorder profiles. These will be subsequently used as an input for the trained TANGLE models to make the prediction. As soon as the submission task is completed, the prediction result will be sent to the user via Email.

**Figure 6:**
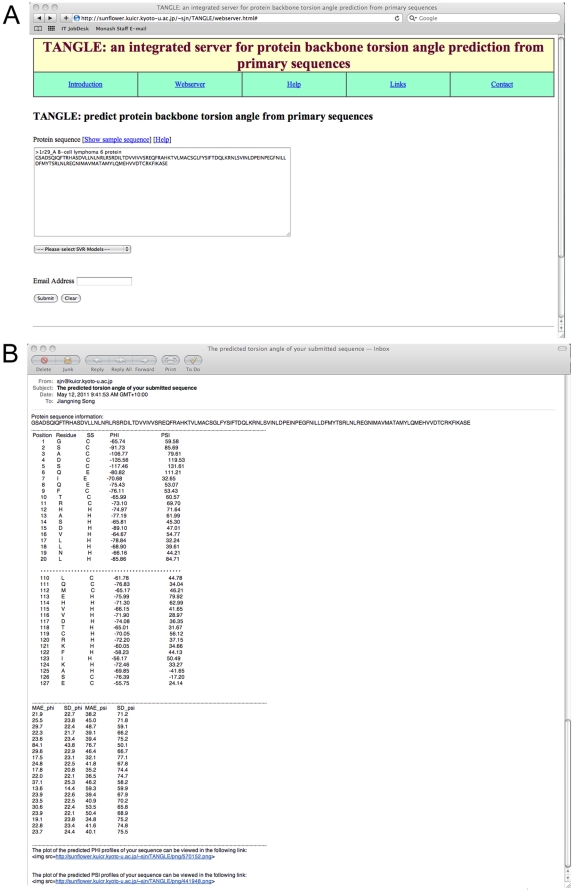
An example of the prediction results by the TANGLE web server. There are two sections: the first section is the primary sequence information of the submitted sequence; in the second section, column 1 is the residue position, column 2 the residue name, while column 3 and 4 correspond to the predicted Phi and Psi angles. In addition, the plots of the predicted Phi and Psi angles are also provided at the bottom of the result webpage.

The TANGLE server is implemented in HTML+Perl and the prediction webpage is shown in Figure 6A. Figure 6B illustrates an example of the prediction results by TANGLE. Basically, there are two sections of the prediction results: the first section is the primary sequence information of the submitted sequence; in the second section, columns 1–4 correspond to the residue position, residue name, the predicted Phi and Psi angles, respectively. Furthermore, the plots of the predicted Phi and Psi angles are accessible by clicking the link at the bottom of the result webpage. To facilitate the method developers, the training dataset, testing dataset, and the calculated Phi/Psi angles for all residues in the training/testing dataset used in this work are downloadable in the links webpage. The TANGLE server is currently hosted by a four-CPU Linux system with 16 GB of main memory. The computational time is mainly dependent on the execution of PSI-BLAST, PSIPRED, SCRATCH and DISOPRED2 programs. A typical job of a sequence with 500 residues will take approximately 5 minutes to accomplish.

### Case study

**Figure 7:**
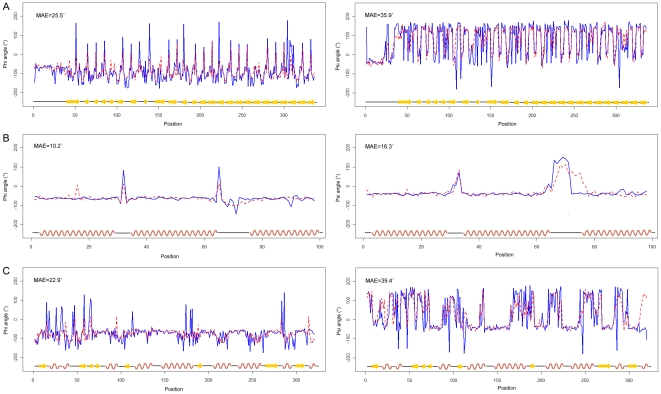
The predicted and observed torsion angles for three typical alpha-, beta-, and alpha/beta-proteins. The three proteins are: (A) the beta1-subunit of the signal-transducing G protein heterotrimer (PDB: 1b9x, chain A) [85]; (B) the enzyme IIAlactose from Lactococcus lactis (PDB: 1e2a, chain A) [86] and (C) the bee venom hyaluronidase (PDB: 1fcv, chain: A) [87]. Secondary structure annotations of these proteins by DSSP [40] are shown at the bottom of each panel, with alpha-helix, beta-strand and coil residues represented by red curves, yellow arrows and black lines, respectively. The observed and predicted torsion angle values are represented by blue-solid and red-dashed lines, respectively.

To understand from where the difficulties of torsion angle prediction arise and illustrate the significance of CC, RMSE and MAE measures used in this study, we presented three illustrative examples of TANGLE prediction of Phi and Psi angles and compared the predicted and observed torsion angle profiles for three proteins (Figure 7): the beta1-subunit of the signal-transducing G protein heterotrimer (PDB ID: 1b9x, chain A) [85], the enzyme IIAlactose from Lactococcus lactis (PDB ID: 1e2a, chain A) [86] and the bee venom hyaluronidase in a complex with hyaluronic acid tetramer (PDB ID: 1fcv, chain A) [87]. To investigate the prediction performance with respect to three secondary structure types, the selected three proteins are classified as beta, alpha, alpha and beta. These are abundant in beta-strands, alpha-helices and mixed with alpha-helices and beta-strands, respectively. The predicted and observed Phi/Psi torsion angles of these three proteins are displayed in Figure 7.

The first example is the beta1-subunit of the signal-transducing G protein heterotrimer with 336 residues and 25 beta-strands [85]. As an all beta-protein, this protein was predicted with a CC of 0.75, a RMSE of 41.6° and a MAE of 25.5° for the Phi angle, and a CC of 0.74, a RMSE of 57.1° and a MAE of 35.9° for the Psi angle. From Figure 7A, we can see that the majority of its regions are in good agreement with the corresponding observed Phi/Psi values, except for several separate positions like residue positions 53, 141, 182, 224 and 306 for the Phi angle, and residue positions 3, 111, 116, 153 and 306 for the Psi angle.

The second example is an all alpha-protein, the enzyme IIAlactose from Lactococcus lactis [86]. It contains 3 alpha-helices with 98 residues. In contrast, this protein was predicted with better accuracy (CC = 0.72, RMSE = 18.0° and MAE = 10.2° for Phi, and CC = 0.77, RMSE = 18.0° and MAE = 16.3° for Psi, respectively). The MAE values of this protein are much better than the first and third examples (See discussion below). Most of the predicted torsion angles are in good agreement with the corresponding observed values. Only the region between residue positions 68 and 73 has the worst prediction with relatively large MAE values (Figure 7B).

The third example is an alpha/beta-protein, the bee venom hyaluronidase. It has 9 alpha-helices, 8 beta-strands, and 320 residues [87]. Compared with the former two examples, it is poorly predicted with a CC of 0.58, an RMSE of 40.9° and an MAE of 21.5° for Phi angle, and a CC of 0.69 and an RMSE of 62.8° and an MAE of 32.4° for Psi angle. The prediction errors, as evaluated by MAEs, are particularly large for residues with the highest or lowest peak torsion angle values (Figure 7C). For this protein, the prediction performance for alpha-helix residues (RMSE = 15.3° and MAE = 13.5° for Phi angle, and RMSE = 80.0° and MAE = 79.5° for Psi angle, respectively) is better than beta-strand (RMSE = 52.4° and MAE = 38.9° for Phi angle, and RMSE = 96.5° and MAE = 91.8° for Psi angle, respectively) and coil residues (RMSE = 57.6° and MAE = 36.7° for Phi angle, and RMSE = 88.3° and MAE = 76.3° for Psi angle, respectively). These results again suggest that the prediction difficulty of torsion angles becomes higher with the increasing degree of irregularity.

## Discussion

Support vector regression (SVR) is a powerful machine learning technique for addressing real-valued prediction tasks in bioinformatics and computational biology, as its strong theoretical basis in statistical learning makes it possible to minimize the generalization error in the prediction [41], [42]. Compared with other traditional techniques, SVR has several advantages such as the handling of data that are non-regularly distributed or have unknown distribution patterns based on kernel functions, the dealing with high-dimensional data, the provision of robust out-of-sample generalization given the approximate choice of parameters, the generation of a solution encompassed by support vectors, the proper balance between bias and variance, etc. Additionally, two-level SVR approach is appropriate for constructing optimal predictors for predicting raw values of samples, as the second-stage predictor is introduced to minimize the generalization error produced in the first stage [55]–[58].

Accurate prediction of protein structural properties such as residue contact number (CN) [5], contact order (CO) [7], solvent accessible surface area (ASA) [9], half-sphere exposure (HSE) [6], residue depth (RD) [8], [16], [73] and so forth can provide valuable information for protein tertiary structure prediction. In previous studies, incorporation of the evolutionary profile in the form of position-specific scoring matrices and predicted structural features such as secondary structure, solvent accessibility and native disorder in the machine learning framework has been shown to be useful for improving the prediction accuracy of protein structural properties. In this study, we have developed a new SVR-based approach TANGLE for the real-valued prediction of protein backbone torsion angles from protein primary sequences. Based on a large benchmark dataset of non-homologous proteins, TANGLE has outperformed an amino acid-specific predictor and one of the state-of-the-art tools ANGLOR [11].

Nevertheless, the further improvement of the prediction accuracy of these structural properties is still a challenging problem. More recently, Ahmad *et al.* proposed novel computational frameworks to predict a variety of structural features of proteins in an integrated manner and the performance of their integrated system was significantly better than that of the models trained separately on individual features [39]. This represents an important step towards developing next-generation of one-dimensional predictors and have important implications in better understanding of how these predictable structural features correlate with each other and collectively dictate the dynamics of the protein structures. In future work, it would be particularly interesting to explore the possibility of applying this integrative framework to develop more accurate predictors and comprehensively compare the integrated models, individual models and two-stage models in terms of computational cost, performance and parameters that need to be optimized.

In general, the Psi angles are more difficult to predict than the Phi angles. We found that the distribution of Phi/Psi angles shows different diversities between different secondary structure types, thereby resulting in different degrees of prediction difficulties. Among the three secondary structure types, the prediction error for alpha-helix residues is the smallest, followed by beta-strand residues, while coil residues have the largest MAE values. Also, the torsion angles of the exposed residues are more difficult to predict than the buried residues. Due to the various degrees of steric collision effects on side-chains with backbones, different amino acids also have different degrees of prediction difficulties. All these results indicate that the training specific predictors for various residue types and secondary structure types might be helpful for the further improvement of the prediction performance. Moreover, incorporation of more relevant features that complement the current feature sets and proper selection of more informative features by powerful feature selection techniques will also be useful for improving prediction accuracy in future. Further improvement can be also achieved by better dealing with the under-represented residues that have less adequate numbers of data points fed into the prediction models. All these issues constitute the subject of future studies.

In this article, we have developed a new approach TANGLE to predict real-valued torsion angles from primary sequences by using a two-stage support vector regression approach. TANGLE used a variety of multiple sequence-derived features, including the evolutionary profiles in the form of position-specific scoring matrices, predicted secondary structure, solvent accessibility and natively disordered region as well as other global sequence features. We have comprehensively assessed the effects of different sequence encoding schemes on the prediction performance of torsion angles. When evaluated based on a large benchmark dataset of 1,526 non-homologous proteins, the prediction performance of TANGLE has been shown to outperform a state-of-the-art predictor ANGLOR and an amino acid-specific predictor. Our work provides a complementary and useful approach towards the more accurate prediction of protein backbone torsion angles and complements the current torsion angle prediction algorithms. We hope that by applying the predicted torsion angles as useful restraints, TANGLE will provide significant assistance in facilitating protein structure prediction and protein fold recognition.

## Supporting Information

Dataset S1 The Phi angles in the training set of 500 protein chains. The first, second, third, fourth and fifth columns in this file correspond to the residue name, the chain name in PDB structures, the original residue position in the PDB ATOM records, the observed Phi angle calculated by DSSP [40], and the normalized Phi angle which will be used as input to TANGLE, respectively. The last three columns correspond to the annotations of secondary structures by DSSP [40], predicted solvent accessibility by SCRATCH [61] and predicted native disorder by DISOPRED2 [62].
(TXT)

Dataset S2 The Psi angles in the training set of 500 protein chains. The description for each column in this file is similar as the above Dataset S1.
(TXT)

Dataset S3 The Phi angles in the testing set of 1,026 protein chains. The description for each column in this file is similar as the above Dataset S1.
(TXT)

Dataset S4 The Psi angles in the testing set of 1,026 protein chains. The description for each column in this file is similar as the above Dataset S1.
(TXT)

Dataset S5 The prediction performance of Phi angle by TANGLE on the testing set. The prediction performance of Phi angle by TANGLE on the testing set of 1,026 protein chains, as evaluated by four measures: CC, RMSE_norm, RMSE_raw and MAE. These measures were calculated at the protein chain level. The first to fourth columns in the file correspond to CC, RMSE_norm, RMSE_raw and MAE, respectively.
(TXT)

Dataset S6 The prediction performance of Psi angle by TANGLE on the testing set. The prediction performance of Psi angle by TANGLE on the testing set of 1,026 protein chains, as evaluated by four measures: CC, RMSE_norm, RMSE_raw and MAE. These measures were calculated at the protein chain level. The description for each column in this file is as the above Dataset S3.
(TXT)
